# Effect of short-term colored-light exposure on cerebral hemodynamics and oxygenation, and systemic physiological activity

**DOI:** 10.1117/1.NPh.4.4.045005

**Published:** 2017-11-20

**Authors:** Felix Scholkmann, Timo Hafner, Andreas Jaakko Metz, Martin Wolf, Ursula Wolf

**Affiliations:** a University of Bern, Institute of Complementary Medicine, Bern, Switzerland; b University of Zurich, University Hospital Zurich, Biomedical Optics Research Laboratory, Department of Neonatology, Zurich, Switzerland

**Keywords:** near-infrared spectroscopy, colored-light exposure, visual stimulation, systemic-physiology-augmented functional near-infrared spectroscopy

## Abstract

There is not yet a comprehensive view of how the color of light affects the cerebral and systemic physiology in humans. The aim was to address this deficit through basic research. Since cerebral and systemic physiological parameters are likely to interact, it was necessary to establish an approach, which we have termed “systemic-physiology-augmented functional near-infrared spectroscopy (SPA-fNIRS) neuroimaging.” This multimodal approach measures the systemic and cerebral physiological response to exposure to light of different colors. In 14 healthy subjects (9 men, 5 women, age: 33.4±10.5 years, range: 24 to 57 years) exposed to red, green, and blue light (10-min intermittent wide-field visual color stimulation; 15×20  s blocks of visual stimulation), brain hemodynamics and oxygenation were measured by fNIRS on the prefrontal cortex (PFC) and visual cortex (VC) simultaneously, in addition with systemic parameters. This study demonstrated that (i) all colors elicited responses in the VC, whereas only blue evoked a response in the PFC; (ii) there was a color-dependent effect on cardiorespiratory activity; (iii) there was significant change in neurosystemic functional connectivity; (iv) cerebral hemodynamic responses in the PFC and changes in the cardiovascular system were gender and age dependent; and (v) electrodermal activity and psychological state showed no stimulus-evoked changes, and there was no dependence on color of light, age, and gender. We showed that short-term light exposure caused color-dependent responses in cerebral hemodynamics/oxygenation as well as cardiorespiratory dynamics. Additionally, we showed that neurosystemic functional connectivity changes even during apparently stress-free tasks—an important consideration when using any of the hemodynamic neuroimaging methods (e.g. functional magnetic resonance imaging, positron emission tomography, and fNIRS). Our findings are important for future basic research and clinical applications as well as being relevant for everyday life.

## 1 Introduction

To understand the impact of light on human physiology, it is necessary to distinguish between visual and nonvisual effects.

Visual effects relate to the processing of the incident light by photoreceptor cells in the retina.^1^ The color is directly quantified by the differential responsivity of three types of cones with sensitivity maxima at ∼560  nm (red), ∼530  nm (green), and ∼420  nm (blue).^2^ From the photoreceptors, the signals are transmitted to bipolar cells, then onto ganglion cells, and finally via the optic nerve to the visual cortex (VC).

Nonvisual effects refer to additional and different types of photoreceptors. One main class includes intrinsically photosensitive retinal ganglion cells (ipRGCs)^3^ with a maximum sensitivity in the blue.^4^ The ipRGCs transmit signals to the hypothalamus, epithalamus, limbic system, and the midbrain,^5^^–^^9^ i.e., brain areas involved in regulating the autonomic nervous system (ANS) and oscillatory physiological processes. It is known that ipRGCs play a fundamental role in regulating chronobiological processes in humans, such as circadian rhythms, sleep, and psychological state.^10^^–^^14^ In particular, melatonin production of the pineal gland is modulated by information from the hypothalamus (i.e., the suprachiasmatic nuclei) based on input received from the ipRGCs about the intensity of blue light.^15^^–^^17^ Knowledge of this mechanism recently triggered numerous studies investigating the potential for disease-promoting effects of blue light on human physiology.^18^^,^^19^ This is relevant since blue light is increasingly prevalent in our environment due to energy-saving light bulbs, computer screens, smartphone screens, etc. The recent discovery that melatonin suppression by blue light has an even lower threshold than previously thought indicates that exposure to colored light may have far-reaching implications for human health.^20^

Many studies show that light evokes different physiological and psychological responses, depending on the color; e.g., emotional^21^^–^^25^ and cognitive^5^^,^^22^^,^^26^^–^^28^ brain activity in humans depends on the color of light exposure. Homeostatic sleep regulation is also affected.^29^ For example, as shown by a famous study,^30^ there is an effect of color on cognitive task performance [“red (versus blue) color primarily induces an avoidance (versus approach) motivation and that red enhances performance on a detail-oriented task, whereas blue enhances performance on a creative task”]. In addition, a recent study^31^ about color-dependent psychological effects found that the type of color affected the perception of interval duration in subjects (“perceived duration was shorter in a red condition than in a blue one”); the effect was also dependent on the type of task.

In a medical context, (colored) light is employed to treat depression,^32^^,^^33^^–^^35^ anxiety,^36^ or seasonal affective disorders.^35^^,^^37^^–^^42^ The increasingly acknowledged significance of light on human physiology will lead to further medical applications, e.g., it is likely that studies will soon emerge exploring the treatment of critically ill adult^43^^,^^44^ and neonatal patients.^45^

Each light stimulus evokes both visual and nonvisual effects, involving the cerebral and systemic physiology. There is, however, not yet a comprehensive view of how the color of a light stimulus affects the cerebral and systemic physiology in parallel. The VC is relatively well investigated,^46^^–^^52^ and research on the cerebral processing of ipRGC-mediated nonvisual effects is increasing.^53^^–^^55^ Despite this, there is a shortage of—and need for—basic research into the visual and nonvisual effects including the human brain (both in the VC and in other brain regions processing visual/nonvisual information) and systemic physiology evoked by a colored-light exposure. The prefrontal cortex (PFC) is of particular interest since it is not only functionally connected to the VC, enabling a higher-order cognitive processing of visual/color information,^56^ but also to the suprachiasmatic nucleus (SCN) in the hypothalamus, which receives information from the ipRGCs via the medial PFC.^57^ It, therefore, makes sense to investigate the PFC’s role in processing colored-light stimuli. In pioneering studies in humans using functional near-infrared spectroscopy (fNIRS), long-term blue light elicited a stronger hemodynamic response in the PFC than red light.^58^ In contrast, color sequences (blue/red, red/blue) showed no significant effect.^59^

In order to reach a comprehensive view of how the color of light affects cerebral and systemic physiology, we established a multimodal simultaneous measurement of response to different colors.

## 2 Materials and Methods

### 2.1 Subjects

Fourteen healthy subjects (9 men, 5 women, age: 33.4±10.5 years, range: 24 to 57 years) were asked not to smoke, eat, or consume any stimulants such as caffeine or energy drinks for 2 h before the start of the measurements. The study was approved by the Ethical Committee of the Canton of Zurich. Informed consent was obtained from the subjects prior to each measurement.

### 2.2 Experimental Protocol and Experimental Setup for Wide-Field Visual Color Stimulation

Following a randomized crossover design, each subject was measured during three different experimental conditions: red, green, or blue. Each task was performed on a separate day to avoid potential carry-over effects but at the same time each day for each individual subject to exclude chronobiological effects. The mean time of measurement was 12:24 with a standard deviation of 2.36 h.

**Fig. 1:**
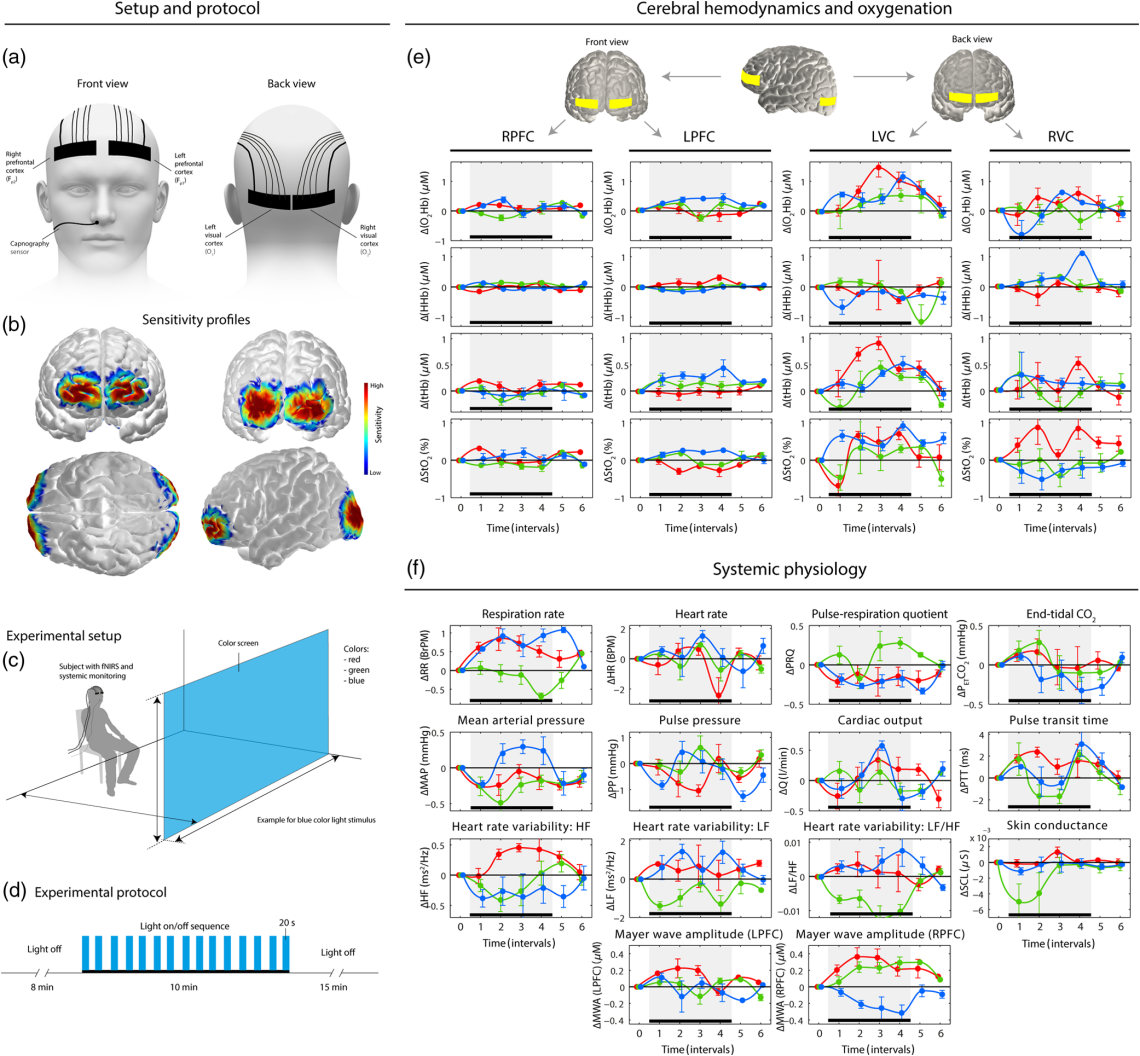
(a) Placements of the NIRS-optode (right and left PFC at positions Fp2 and Fp1; right and left VC at positions $O_{2}$ and $O_{1}$), (b) sensitivity profile of NIRS-sensors on the brain, (c) experimental setup with position of the subject and the color screen, and (d) experimental protocol. (e) and (f) Overview of the block-averaged (group-level) stimulus-evoked changes in cerebral hemodynamics/oxygenation and systemic physiology (median±standard error of the median). Black bar and gray area: time interval during visual stimulation.

Each measurement lasted 33 min, i.e., 8-min baseline in darkness (interval 1), 10-min intermittent wide-field visual color stimulation (interval 2), and 15-min recovery in darkness (interval 3). The visual stimulation followed an event-related design of 15 alternating light-on (20 s) and light-off periods. The length of the light-off periods varied randomly [mean 21.1±3.3  s, range 17 to 27 s, Fig. 1(d)] to prevent habituation. During the measurements, the subjects sat opposite a screen (width: 2 m, height: 3 m, distance from subject to screen: 1.2 m) illuminated by four light-emitting diode beams (PAR 56 CAN RGB 05 BS, Cameo, Adam Hall GmbH, Neu-Anspach, Germany), which created a homogenous color. They were reproducibly controlled by a DMX mixer. The illuminance of the light at the eye level of the subjects was 20 lx for each color as measured by the LT300 luminance meter (Extech Instruments, Nashua, New Hampshire). The wavelengths of the maximum intensity determined by the MAYA 2000-Prospectrometer (Ocean Optics, Inc.) were 682 nm (red), 515 nm (green), and 465 nm (blue).

Subjects were instructed not to move their body or head during the measurement to prevent movement artifacts (MAs).

### 2.3 Systemic-Physiology-Augmented Functional Near-Infrared Spectroscopy: Equipment

The following devices were applied to simultaneously assess changes in cerebral hemodynamics and oxygenation, and systemic physiological activity: (i) a multichannel frequency-domain NIRS system (Imagent, ISS Inc., Champaign, IL), (ii) a gas analyzer (Nellcor N1000, Covidien, Dublin, Ireland), (iii) a continuous noninvasive blood pressure (NIBP) monitor (SOMNOtouch, Somno Medics, Randersacker, Germany), and (iv) a skin conductance measuring device (Mind-Reflection, Audiostrobe Ltd., UK).

The ISS Imagent frequency-domain NIRS system determined with 50-Hz resolution absolute values of the tissue oxygen saturation (${\mathrm{StO}}_{2}$), oxyhemoglobin ($\left[O_{2}\mathrm{Hb}\right]$), deoxyhemoglobin ([HHb]), and total hemoglobin ([tHb]) concentration by the frequency-domain multidistance (FDMD) method^60^ calculated by the ISS software. The Imagent employs 16 laser diodes at 834 nm and 16 at 760 nm, and 4 highly sensitive photomultiplier tubes as detectors. Optodes 1 and 2 had source–detector separations of d=2.0, 2.5, 3.5, and 4.0 cm. Optodes 3 and 4 had d=2.5, 3.0, 3.5, and 4.0 cm. Their multidistance geometry minimized the influence of extracerebral tissue such as scalp^61^^,^^62^ and reduced MAs.^63^ The correlation coefficient of the slopes enables identifying tissue inhomogeneities.^64^ Optodes 1 and 2 were placed over the left (${\mathrm{Fp}}_{1}$) and right (${\mathrm{Fp}}_{2}$) PFC (LPFC and RPFC, respectively), and optodes 3 and 4 over the right ($O_{2}$) and left ($O_{1}$) VC (RVC and LVC, respectively) [Fig. 1(a)] according to the international 10 to 20 system^65^ based on manually determined positions of standard landmarks (inion, nasion, and periauricular points). The latter covered the primary VC (V1) and the secondary VC (V2) [Fig. 1(b)]. The attachment of the opcodes to the head was done is such a way to avoid any discomfort and stress for the subjects, i.e., attaching the optodes too tightly was avoided, and it was ensured that no sharp edges of the optodes caused discomfort or pain.

A Nellcor N1000 gas analyzer measured the partial pressure of exhaled ${\mathrm{CO}}_{2}$ ($P_{\mathrm{ET}}{\mathrm{CO}}_{2}$) noninvasively with a probe positioned directly below the right nostril of the subject [Fig. 1(a)].

The SOMNOtouch continuous NIBP device measured and determined the following parameters: mean arterial blood pressure (MAP), systolic blood pressure (SBP), diastolic blood pressure (DBP), pulse pressure (PP), pulse transit time (PTT), heart rate (HR), high-frequency (HF) (0.15 to 0.4 Hz) component of the HR variability (HF-HRV), and the low-frequency (LF) (0.04 to 0.15 Hz) component of the HRV (LF-HRV).

To determine the status of the ANS, the skin-conductance biofeedback device measured the electrodermal activity (EDA) and the skin conductance level (SCL) at 2 Hz. Electrodes were attached to the distal phalange of the index and middle fingers of the right hand.

The methodical approach employed in our study of measuring changes in systemic physiology as well as local perfusion and oxygenation in the head we termed “systemic-physiology-augmented functional near-infrared spectroscopy” (SPA-fNIRS). The term refers to use systemic physiology measurements in order to assist, complement, improve, i.e., augment, the “traditional” fNIRS measurements.

### 2.4 Assessment of Psychological Changes

The mood state of the subjects before and after the experiment was determined by the multidimensional mood questionnaire (MDBF),^66^ yielding the good or bad mood (GS), the vigilance (WM), and the nervousness (RU) of the subject. For these three parameters, the postexperiment minus preexperiment differences were calculated (ΔGS, ΔWF, and ΔRU). Deviations from a median of zero and influences of the subject’s gender and light color were assessed by a Wilcoxon signed rank test. The influence of age was tested using linear regression analysis.

### 2.5 Signal Preprocessing

All signal preprocessing was performed in MATLAB (R2013b, MathWorks, Natick). MAs in the ${\mathrm{StO}}_{2}$, $\left[O_{2}\mathrm{Hb}\right]$, [HHb], and [tHb] signals were removed by the movement artifact reduction algorithm (MARA).^67^ The most prevalent MAs were spikes and sudden baseline shifts. In total, 3.3% of the fNIRS signal time was defined as artifacts, which were corrected by applying the MARA approach. MARA was evaluated as a useful^68^^–^^71^ or moderately useful^72^^,^^73^ MA correction technique. All parameters for MARA were selected for each signal separately and optimized to obtain an effective removal of MAs without disturbing the signal.

The fNIRS signals were downsampled to 2 Hz to remove high-frequency physiological and measurement noise by applying a low-pass filter to prevent aliasing. To further remove high-frequency noise, a moving average with a span of 2.4 s was applied.

The capnography signal was resampled to 2 Hz and the envelope calculated, from which the $P_{\mathrm{ET}}{\mathrm{CO}}_{2}$ was determined. To remove high-frequency noise, a robust local regression using weighted linear least squares and a second degree polynomial model (RLOES) with a bin of 20 samples were applied. This approach removes random noise while preserving the physiologically relevant high-frequency content. $P_{\mathrm{ET}}{\mathrm{CO}}_{2}$ is directly related to the arterial ${\mathrm{pCO}}_{2}$ (${\mathrm{PaCO}}_{2}$).^74^ The respiration rate (RR) was extracted from the capnography signal by (i) detecting the maxima of every breath employing our peak detection algorithm,^75^ (ii) calculating the time differences between successive peaks (ΔT), and (iii) resampling ΔT to obtain an equidistantly sampled signal of the RR by a piecewise cubic Hermite interpolating polynomial. In addition, to quantify the coupling between RR and HR, the pulse-respiration quotient (PRQ) was calculated (PRQ=HR/RR).

All other systemic physiology data, except the skin conductance, were also denoised by the RLOES method.

From the LF- and HF-HRV signals, the LF/HF ratio (LF/HF) was calculated. Furthermore, the cardiac output (Q) was calculated according to Q=(PP×HR)/(SBP+DBP).^76^

### 2.6 Signal Processing and Statistical Data Analysis

For the final analysis, the following signals were included: (i) fNIRS-signals, i.e., $\left[O_{2}\mathrm{Hb}\right]$, [HHb], [tHb], ${\mathrm{StO}}_{2}$, and Mayer wave amplitude (MWA), and (ii) systemic physiological signals, i.e., HR, RR, PRQ, $P_{\mathrm{ET}}{\mathrm{CO}}_{2}$, MAP, PP, Q, PTT, SCL, HF, LF, and LF/HR.

To assess the changes elicited by the light stimulation, (i) stimulus-evoked changes were block averaged and (ii) neurosystemic functional connectivity was analyzed. Statistical significance was evaluated at subject and group level.

For the block averaging, each of the segments had a length of 35 s starting 5 s before the stimulus. Each single trial was normalized to the baseline value and linearly detrended. Then, the medians in the intervals 1 to 4, 6 to 9, 11 to 14, 16 to 19, 21 to 24, 26 to 29, and 31 to 34 s were calculated. Thus, the stimulus period was covered by four intervals between 6 and 24 s. For each trial, subject, and signal, it was tested whether the block-averaged stimulus-evoked changes were significant by a Wilcoxon signed rank test (subject-level analysis). False discovery rate (FDR) correction for multiple comparisons was applied. The p and h values of the statistical tests were stored for the subsequent group level statistical analysis. Here, all block averages of the stimulus-evoked changes for each subject and task were analyzed by quantile regression models using the R quantreg-package.^77^ Quantile regression is more robust against outliers and does not assume a normal distribution of errors compared to mean and least squares regressions. Confidence intervals for the regression were calculated by bootstrapping^78^ with 100,000 bootstraps. It was tested whether the stimulus-evoked changes were significant and whether they depend on the factors color of light, age, and gender. The fNIRS signals were separately analyzed for the VC and PFC, but left and right VC or PFC were combined to increase the power of the statistics.

The inputs for the quantile regression were the block averages of each subject and task, where nonsignificant changes in the single block averages were weighted by zero. This step ensured that only statistically significant subject level data were included in the group-level analysis, thus reducing intersubject variability that confounds the potential color-type dependency.

To analyze the neurosystemic functional connectivity, for each person and each trial, a correlation matrix was computed by comparing each block average of each signal to any other signal by the Spearman correlation coefficient ($r_{s}$) and its statistical significance (p<0.05, with FDR correction for multiple comparisons). If the correlation was not statistically significant, the value was deleted in the matrix. Then, the correlation matrices for all trials and subjects were averaged. Additionally, matrices were calculated only for the fNIRS signals and only for the systemic physiological signals. Then, all correlation matrices were analyzed by transforming the respective matrix into a network where the nodes represent the block-averaged stimulus-evoked signals and the edges represent the connection strengths in terms of the correlation coefficients. The complex network properties, i.e., the assortativity (i.e., the weighted assortativity coefficient, $r^{w}$), transitivity (i.e., the weighted transitivity, $T^{w}$), density (D), and efficiency (i.e., the weighted global efficiency, $E^{w}$) were calculated using the MATLAB Brain Connectivity Toolbox.^79^ The complex network parameters for each color were compared using a nonparametric Wilcoxon rank sum test.

Each complex network parameter is related to specific properties of the network created by the correlation matrices.^79^ $r^{w}$ quantifies the correlation strength between the strengths of all nodes of the network at two opposite ends of a link. The higher the value, the higher the number of nodes in the network, which tend to link to other nodes with the same or similar strength. $T^{w}$ is associated with the degree to which nodes in a network tend to cluster together. It is a classical version of the clustering coefficient. The higher the value, the higher the prevalence of clustered connectivity around individual nodes. D is a measure of the mean network degree, i.e., the relation between the potential connections in a network with the actual connections. A high value indicates that many nodes are connected with each other. Finally, $E^{w}$ is related to the average inverse shortest path length between all pairs of nodes in the network. It is inversely related to the characteristic path length. The higher the value, the more interconnected the nodes are.

## 3 Results

### 3.1 Changes During All Three Color Stimuli

The results are depicted in Figs. 1(e) and 1(f).

#### 3.1.1 Cerebral tissue hemodynamics and oxygenation

All three color stimuli evoked statistically significant changes in $\left[O_{2}\mathrm{Hb}\right]$, [HHb], [tHb], and ${\mathrm{StO}}_{2}$ in the VC, i.e., increases in $\left[O_{2}\mathrm{Hb}\right]$, [tHb], and ${\mathrm{StO}}_{2}$, and a decrease in [HHb] with the exception of [HHb] and ${\mathrm{StO}}_{2}$ during green color.

In the PFC, only the blue color elicited significant changes, i.e., an increase in $\left[O_{2}\mathrm{Hb}\right]$, [tHb], and ${\mathrm{StO}}_{2}$.

#### 3.1.2 Cardiorespiratory activity

The stimulus-evoked changes in cardiorespiratory parameters generally showed a large intersubject variability. At group-level, the following parameters elicited statistically significant changes [Fig. 1(f)]: PRQ (decrease during blue-light exposure), PTT (increase during blue), MAP (decrease during red and green), RR (increase during blue and red), HR (increase during blue), HF (increase during red), LF/HF (decrease during green), and MWA (RPFC) (increase during red and green). No statistically significant changes were observed for $P_{\mathrm{ET}}{\mathrm{CO}}_{2}$, Q, PP, LF, and MWA (LPFC).

#### 3.1.3 Electrodermal activity

Again, there was large intersubject variability. Green-light exposure showed a nonsignificant trend toward a decrease in the SCL [Fig. 1(f)].

#### 3.1.4 Neurosystemic functional connectivity

Stimulus-evoked changes in cerebral parameters and systemic physiology correlated statistically significantly for seven pairs of variables: $\left[O_{2}\mathrm{Hb}\right]$ (RVC) versus MWA (RPFC), $r_{s}=0.505\pm 0.474$; [HHb] (LPFC) versus LF, $r_{s}=0.424\pm 0.383$; [tHb] (LPFC) versus MWA (RPFC), $r_{s}=-0.399\pm 0.419$; Q versus MWA (RPFC), $r_{s}=-0.379\pm 0.440$; [HHb] (RVC) versus MWA (RPFC), $r_{s}=-0.358\pm 0.393$; [HHb] (LVC) versus HF, $r_{s}=0.337\pm 0.374$; and [HHb] (LVC) versus Q, $r_{s}=-0.311\pm 0.320$. This demonstrates the neurosystemic functional connectivity.

Additionally, functional connectivity was shown between the cerebral signals: [HHb] (LPFC) versus ${\mathrm{StO}}_{2}$ (LPFC), $r_{s}=-0.921\pm 0.093$; [HHb] (RPFC) versus ${\mathrm{StO}}_{2}$ (RPFC), $r_{s}=-0.920\pm 0.088$; [HHb] (RVC) versus ${\mathrm{StO}}_{2}$ (RVC), $r_{s}=-0.916\pm 0.099$; [HHb] (LVC) versus ${\mathrm{StO}}_{2}$ (LVC), $r_{s}=-0.904\pm 0.145$; $\left[O_{2}\mathrm{Hb}\right]$ (RVC) versus ${\mathrm{StO}}_{2}$ (RVC), $r_{s}=0.846\pm 0.087$; $\left[O_{2}\mathrm{Hb}\right]$ (LPFC) versus [tHb] (LPFC), $r_{s}=0.820\pm 0.107$; and $\left[O_{2}\mathrm{Hb}\right]$ (LVC) versus ${\mathrm{StO}}_{2}$ (LVC), $r_{s}=0.813\pm 0.178$. Relatively strong and nontrivial correlation between systemic physiology signals were observed for the following pairs: PP versus PTT, $r_{s}=-0.607\pm 0.199$; MAP versus PTT, $r_{s}=-0.550\pm 0.212$; PP versus Q, $r_{s}=0.537\pm 0.208$; $P_{\mathrm{ET}}{\mathrm{CO}}_{2}$ versus MAP, $r_{s}=0.475\pm 0.400$; HR versus PP; $r_{s}=-0.416\pm 0.363$; $P_{\mathrm{ET}}{\mathrm{CO}}_{2}$ versus PP, $r_{s}=0.410\pm 0.310$.

**Fig. 2:**
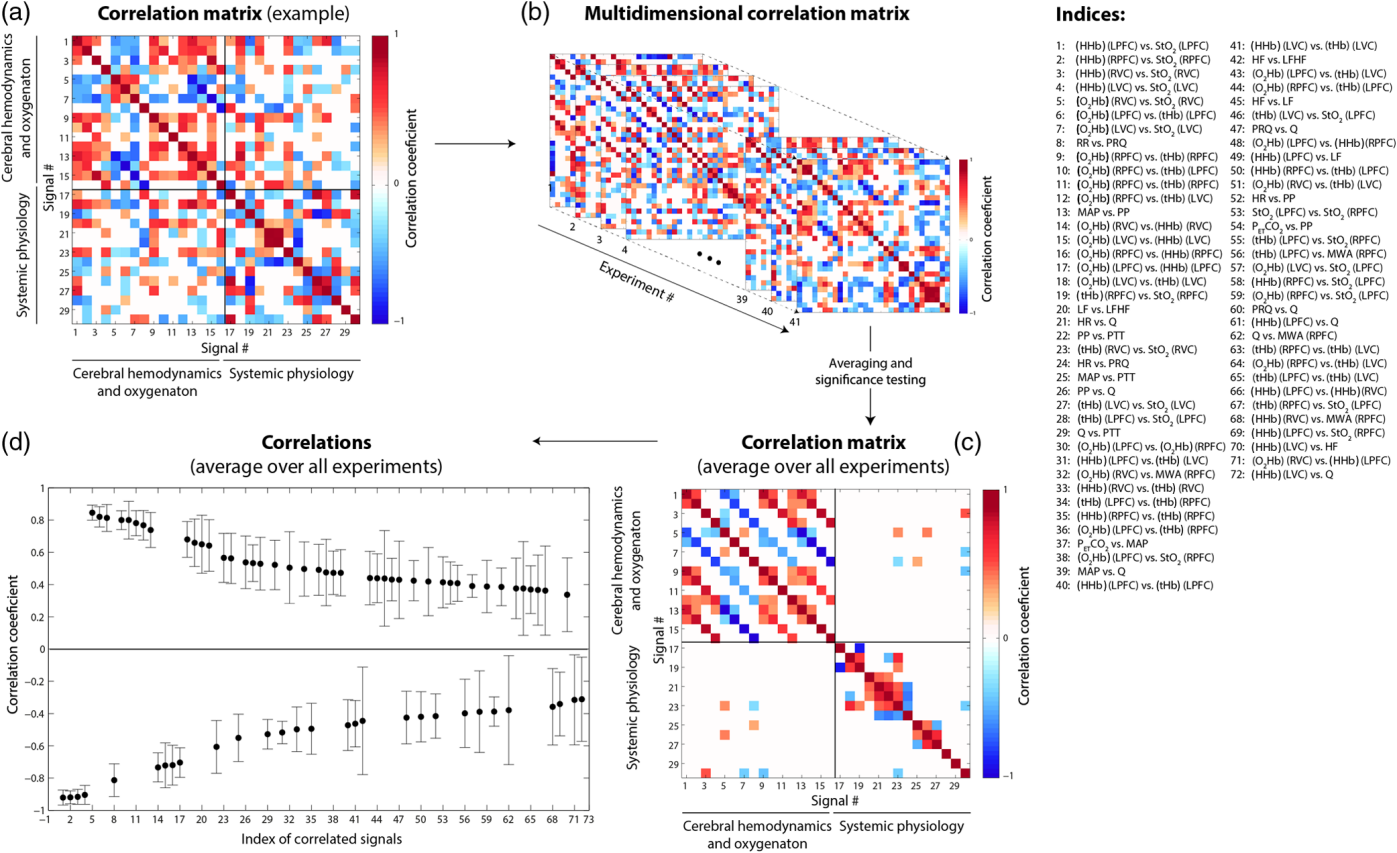
Visualization of the correlation analysis. (a) Exemplary correlation matrix of one subject and trial. (b) Combining the correlation matrices of each subject and trial results in a multidimensional correlation matrix. (c) Averaging these 3-D matrices generates the group-averaged correlation matrix. (d) Statistical analysis identifies the significant correlations shown here.

All 72 significant correlations are visualized in Figs. 2(c)–2(d).

### 3.2 Dependence of Changes on Color of Light, Gender, and Age

#### 3.2.1 Cerebral parameters

${\mathrm{StO}}_{2}$ was significantly color dependent in the VC (the responses to blue compared to green, as well as to red compared to green were different). This color-dependent effect was even stronger in the PFC for blue versus red and green, both in ${\mathrm{StO}}_{2}$ and [HHb]. No color dependence was observed for $\left[O_{2}\mathrm{Hb}\right]$ and [tHb].

Gender had a significant effect on the [HHb] and ${\mathrm{StO}}_{2}$ responses in the PFC (tested with combined values from the left and right PFC), and $\left[O_{2}\mathrm{Hb}\right]$, [HHb], and ${\mathrm{StO}}_{2}$ responses in the VC.

Age was significantly negatively correlated with the magnitude of stimulus-evoked changes in ${\mathrm{StO}}_{2}$ of the PFC.

#### 3.2.2 Cardiorespiratory activity

Color had a significant effect on the magnitude of the changes in RR (green versus red and blue), PRQ (green versus red and blue), HF (red versus blue and green), LF (red versus blue and green), LF/HF (blue versus red and green, with green light leading to a decrease), and MWA (RPFC) (blue versus red and green).

Gender was a discriminating factor for the changes in RR, HR, PRQ, $P_{\mathrm{ET}}{\mathrm{CO}}_{2}$, Q, PP, and MWA (RPFC).

Age was significantly correlated with the magnitude of stimulus-evoked changes in PRQ, RR, LF, LF/HF, and MWA (LPFC). There was a larger magnitude of changes in younger subjects for RR, LF, LF/HF, and a smaller one for PRQ and MWA (LPFC).

#### 3.2.3 Electrodermal activity

SCL was not observed to depend on color of light, age, or gender, probably due to a large intersubject variability.

#### 3.2.4 Neurosystemic functional connectivity

**Fig. 3:**
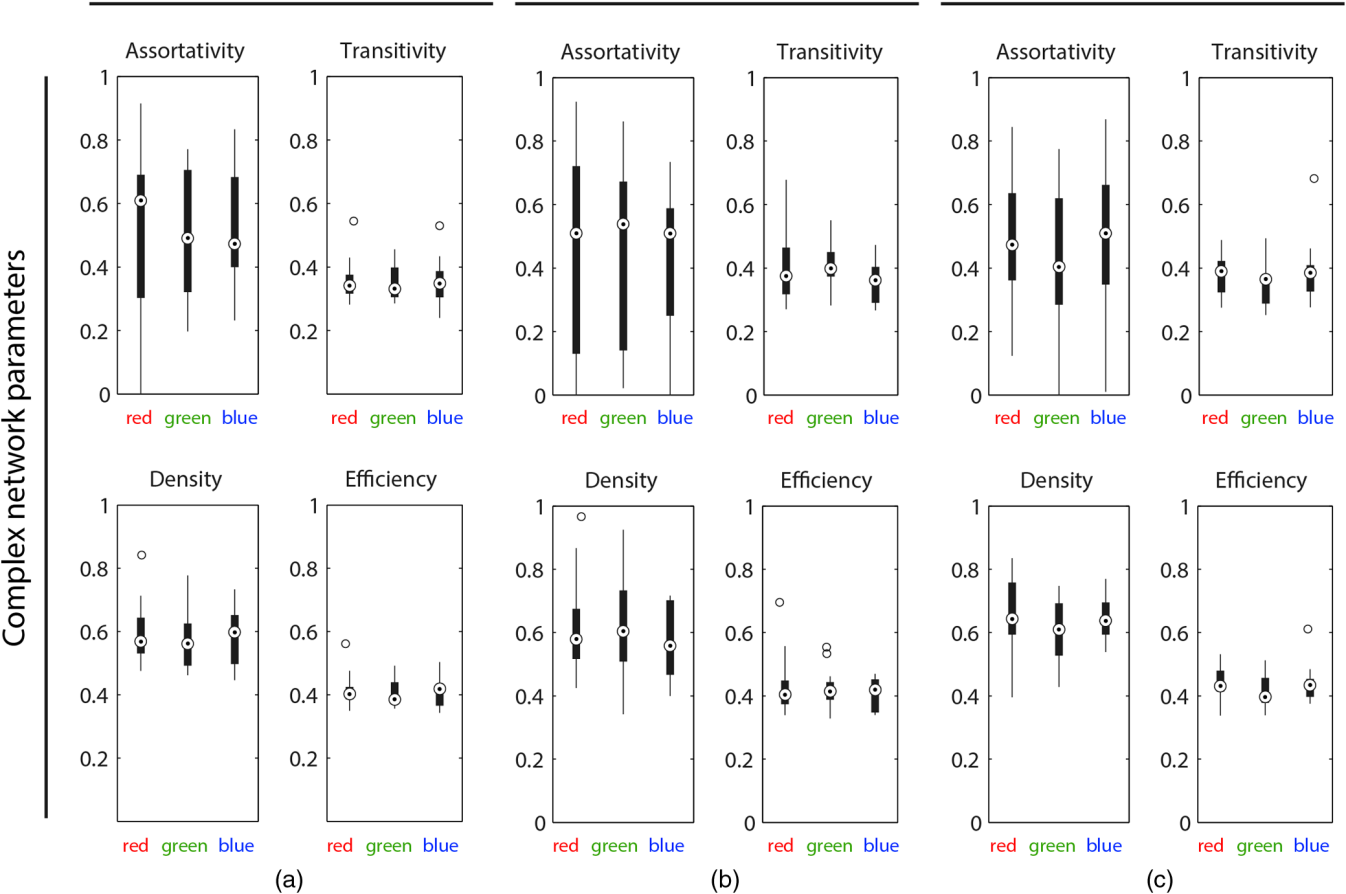
Results of the complex network analysis. The values for the assortativity, transitivity, density, and efficiency were plotted for the three colors and the three types of correlation analyses performed: (a) cerebral hemodynamics/oxygenation + systemic physiology (i.e., eurosystemic functional connectivity), (b) cerebral hemodynamics/oxygenation, and (c) systemic physiology.

Figure 3 shows the results for the complex network parameters ($r^{w}$, $T^{w}$, D, and $E^{w}$). The parameters were not found to show significant color dependence.

#### 3.2.5 Psychological state

Changes in the subjects’ psychological state (ΔGS, ΔWF, and ΔRU) following color stimulation did not show any significant correlation with the color of light, age, time of measurement, or gender.

## 4 Discussion

### 4.1 Cerebral Tissue Hemodynamics and Oxygenation

The results show that the two cortices assessed, i.e., PFC and VC, react differently to short-term color stimuli. While the hemodynamic responses in the VC were stronger than in the PFC, independent of color, the PFC showed a significant hemodynamic response only to blue. These differing responses in the two cortices reflect different underlying processes.

#### 4.1.1 Color-independent response of the VC

The observation that the magnitude of the hemodynamic responses in the VC was independent of color (except for the color-specific response in ${\mathrm{StO}}_{2}$) is in agreement with an functional magnetic resonance imaging (fMRI) study (V1; red, green, yellow, and blue; duration of stimulation: 12 s; luminance: $470\;\mathrm{cd}/m^{2}$).^46^

In an fNIRS study, color pairing was a significant predictor for the magnitude of the hemodynamic response.^80^ The larger the chromaticity separation in the perceptual uniform color space (CIE 1976 UCS), the larger the hemodynamic response in V1. Since in our study the chromaticity separation was similar for the three colors employed as stimulus and the dark interstimulus interval, it is reasonable that the hemodynamic responses to the different colors were similar. Thus, our findings in the VC are in line with the previous literature.^46^^,^^47^^,^^80^^,^^81^

The larger variability of the evoked hemodynamic responses at the VC compared to the PFC [indicated by larger error-bars in the block averages, Fig. 1(e)] might either be due to a lower signal-to-noise-ratio of the measurements at the VC, due to the hair or due to a real larger intersubject variability of the hemodynamic response at the VC compared to the PFC. Analysis of the individual (subject-specific) evoked hemodynamic responses at the VC revealed that the latter is certainly relevant, i.e., a significant intersubject variability of the hemodynamic changes (with respect to the type of waveform as well as magnitude) was observed (data not shown). A detailed analysis of this aspect is planned for future research.

#### 4.1.2 Color-dependent response of the PFC

Our finding that, compared to red and green light, blue caused a significantly different response in $\left[O_{2}\mathrm{Hb}\right]$, [tHb] and ${\mathrm{StO}}_{2}$ in the PFC is of particular interest. First, our result is in line with neurophysiological and chronobiologic findings on the nonvisual effects of blue light. Furthermore, to best of our knowledge, there are very few studies (only four to date, all employing fNIRS) on the response of the PFC to colored light.

The response of the left PFC to a wide-field stimulation with two color sequences, i.e., 5-min blue-light exposure followed by 5-min red-light exposure, or vice versa, was studied in Ref. 59. There was no significant difference between the two sequences, even though blue/red seemed to evoke a larger response compared to red/blue. In a higher powered study, this effect may have reached significance. A direct comparison of this study with our findings is not possible due to the different color stimulation employed, i.e., color sequence versus single color, and long-term (5 min) versus short-term (20 s) color exposure.

In a subsequent study by the same authors,^58^ the effect of a long-term (10 min) wide-field colored-light (red or blue) exposure on the left PFC was assessed. There was no change in [tHb]. A significant increase in ${\mathrm{StO}}_{2}$ was shown during and after exposure to blue but not red light. This is in line with our current findings. It is important that the colored-light stimuli have equal luminescence, as in our current study, because the interaction of color and luminance is nonlinear and complex for the evoked brain activity and the perceived visual impression.^82^^,^^83^

During a cognitive test on a computer screen, the [tHb] in the PFC depended on the background color (white or blue) of the screen.^84^ This study lacks power, because data of only one subject were presented, and without any statistics. Additionally, since only [tHb] was reported, we would be reluctant to even assume a hemodynamic response. Since the superficial signals (scalp) were not removed, it is unclear whether the changes in [tHb] originate from the brain.

Haigh et al.^80^ studied the effect of visual stimuli of horizontal dual-colored gratings with different color combinations on cerebral hemodynamic responses. The primary region of interest was the VC, but one channel was also placed over the PFC. The “small signal” from the PFC did not justify analysis. Thus, it is impossible to compare this to our data.

As mentioned above, the stronger hemodynamic response to blue light is in line with neurophysiological and chronobiological findings. The main reason for this seems to be the blue-light-sensitive photopigment melanopsin in the ipRGCs. It has an action spectrum with a maximum at 484 nm^4^—close to the 465 nm of our study. IpRGCs communicate to the hypothalamus (i.e., SCN, ventro-lateral preoptic nucleus, lateral hypothalamic area, thalamic intergeniculate leaflet, and thalamic lateral geniculate nuclei), the epithalamus (i.e., the lateral habenula, which forms the epithalamus together with the pineal gland), the limbic system (i.e., the amygdala), and the midbrain (i.e., the olivary pretectal nuclei and superior colliculus).^5^^–^^9^ It was shown that the SCN reports by multisynaptic connections to the medial PFC,^57^ forming a pathway that modulates higher-level cognitive functions. Also the amygdala, as part of the limbic system, is connected to the PFC.^85^ The PFC, particularly the medial part, acts as a coordinator for behavioral as well as physiological (autonomic and neuroendocrine) responses to stress.^86^ With our fNIRS setup used in this study, we were also sensitive to the medial PFC as proven by the Monte Carlo simulation of photon-propagation resulting in the sensitivity profile shown in Fig. 1.

Concerning the ANS, specific regions of the PFC are linked to the regulation of the sympathetic nervous system (SNS), i.e., the prelimbic PFC (plPFC) acts as an inhibitor of the SNS and the infralimbic PFC (ilPFC) as a driver of the SNS.^86^^,^^87^ The primary neuroendocrine stress response, represented by the hypothalamic-pituitary-adrenocortical (HPA) axis, is regulated by both the plPFC and ilPFC.^86^^,^^88^^,^^89^ In humans, the right PFC in particular is involved in regulation of autonomic and endocrine responses to stress.^90^^–^^92^

These findings suggest the following pathway: (i) blue light is perceived by the ipRGCs, activating dedicated brain regions such as parts of the hypothalamus (particularly the SCN), epithalamus, limbic system, and midbrain, (ii) light-induced activity of the SNS and limbic system evokes PFC activity, and (iii) PFC activity induces changes in the activity of the ANS and HPA axis.

In addition, other nonvisual pathways may contribute. Photoreceptors located in the human skin may be involved, e.g., the four opsins OPN1 (cone opsin), OPN2 (rod opsin), OPN3 (panopsin or encephalopsin), and OPN5 (neuropsin) expressed by melanocytes and keratinocytes.^93^ In our study, a small amount of skin (face, hands, and parts of the underarm) was also exposed. Whether this exposure might have contributed to the measured physiological responses cannot be determined since it is yet unclear if and how a skin exposure with colored light would have influenced the physiological parameters that were included in our study. There are photoreceptors located in the brain itself, i.e., OPN3,^94^ OPN4,^95^ and OPN5.^96^ The functional role of these latter opsins has not yet been studied so their implication for the present study is unclear. But their presence in brain tissue may indicate a direct nonvisual detection of environmental light.

The functional connections of the PFC with the VC^56^ may contribute, too. For example, the ventral pathway of visual information processing involves the PFC, enabling long-range coupling between the frontal cortex, especially the frontal eye field, and the VC (in particular V4) during attention.^97^ The coupling of the VC with the PFC may represent another route for the color-specific PFC activity shown in our study.

### 4.2 Cardiorespiratory Activity

Color-specific effects in the cardiovascular system and the respiratory system were identified for RR, PRQ, HF, LF, LF/HF, and MWA (RPFC).

There were strong increases in RR for blue and red light and a decrease for green. A negatively correlated change in $P_{\mathrm{ET}}{\mathrm{CO}}_{2}$ would have been expected and such a trend, although nonsignificant, is visible in Fig. 1(f). This negative correlation is well known, i.e., for a higher RR more ${\mathrm{CO}}_{2}$ is exhaled and $P_{\mathrm{ET}}{\mathrm{CO}}_{2}$ falls (or vice versa).^98^ An increase in RR and decrease in $P_{\mathrm{ET}}{\mathrm{CO}}_{2}$ is a typical pattern during an increase in arousal^99^ but is not directly linked to the emotional state.^100^ However, during psychological stress (i.e., negative valence with high arousal), characteristic changes in RR and/or respiratory tidal volume take place.^101^^,^^102^ The arousal-inducing effect of blue light was also shown by EEG:^103^ A 135-s exposure to light reflected from blue paper elicited a stronger decrease in the α attenuation coefficient than light reflected from red paper.

Blue light elicited a strongly significant decrease in PRQ (=HR/RR) compared to green, which can be explained mostly by an increase in RR (Fig. 2). An opposite effect was observed by Gerard^104^ in long-term light exposure of 10 min. Previously, a 2-h exposure to blue light was shown to increase HR,^105^ whereas a 5-min exposure to blue, red, and white light decreased HR.^106^ Thus, the temporal structure of the exposure may be relevant.

Concerning HRV, red light elicited an increase in HF and green a decrease in LF/HF. Traditionally, the LF is considered to be related to the sympathetic and the HF to the parasympathetic part of the ANS.^107^^,^^108^ But in fact both components are influenced by the sympathetic and parasympathetic ANS in parallel,^109^^–^^111^ and the LF/HF ratio does not relate directly to the sympathovagal balance.^111^ The LF component and thus LF/HF are also affected by changes in respiration.^112^ The interpretation of HF and LF/HF by themselves is, therefore, not straightforward. An increase in LF/HF was reported for red and blue light, albeit nonsignificantly for blue during 10-min exposure.^113^ These results are not directly comparable to our study due to the much longer exposure.

The age-dependent effects observed for the stimulus-evoked changes in the systemic parameters [RR, PRQ, LF, LF/HF, and MWA (LPFC)] are expected due to the age dependence of cardiovascular reactivity,^114^^–^^116^ activity and reactivity of the ANS,^117^^–^^120^ and respiration patterns and reactivity.^121^^,^^122^ Gender effects found for RR, HR, PRQ, $P_{\mathrm{ET}}{\mathrm{CO}}_{2}$, PP, Q, and MWA (RPFC) are also known for the cardiovascular reactivity,^116^^,^^123^^–^^127^ activity and reactivity of the ANS,^128^^,^^129^ and respiration patterns and reactivity.^121^^,^^122^^,^^130^

In a follow-up paper, we will report the individual responses in systemic physiological signals observed in the experiment, highlighting that the magnitude and sign of them depends strongly on the individual subject and experimental trial.

### 4.3 Electrodermal Activity

It is rather unexpected that stimulus-evoked changes in EDA were not related to color of light, age, or gender, given the strong correlation of EDA with attention, arousal and emotion,^131^^–^^135^ and the expected color-dependent differences in these psychological factors. This absence may be explained by the fact that we investigated the trend of the EDA, i.e., the tonic SCL and not the rapidly varying changes, i.e., the skin conductance response (SCR). Both components constitute the EDA signal and are related to the sympathetic activity of the ANS.^136^ SCL showed a high intersubject variability, possibly due to different individual psychological states and traits, thus potentially preventing the detection of significant correlations.^137^^–^^143^ In the future, we recommend applying methods to reduce this intersubject variability.^144^

Previous studies found a decrease in the SCL during a 10-min blue-light exposure,^104^ a stronger SCR response to red or green light of 1 min compared to yellow or blue,^145^ and a stronger SCR for red compared to green light.^146^ Interestingly, there were also individual subjects who showed the opposite effect compared to the group.

### 4.4 Neurosystemic Functional Connectivity

Although there were several parameters with significant neurosystemic functional connectivity, these were not color dependent. Color dependence, although present in the PFC and in specific systemic physiological signals (RR, HR, PRQ, MAP, HF, LF/HF, and MWA), differs substantially between individuals and is, therefore, masked. To the best of our knowledge, our study is the first ever to assess the neurosystemic functional connectivity.

### 4.5 Psychological State

Our study was the first to use the MDBF to determine psychological changes associated with colored-light exposure. The psychological state was not affected by the color of light, time of measurement, age, or gender. One possible reason why no correlation was found might be that the MDBF assessment was performed before and after the experiment, i.e., >15  min before/after the light exposure. Any color-specific effect may have been more prominent during the light exposure. Thus, our finding does not rule out a possible color-dependent effect.

Previously, several color-dependent psychological effects were reported: a lower anxiety state was found for blue and green compared to red and yellow wide-field stimulation in healthy subjects.^147^ Red and green light (duration: 1 min) elicited a stronger SCR, indicating arousal, than yellow or blue.^145^ The subjective rating of pleasurableness of a color is a nonlinear function of its intensity and saturation.^148^ Colors in the green–blue spectral region were rated as more pleasant than those in red–yellow.

### 4.6 Strengths and Limitations

#### 4.6.1 Strengths

This study has a number of important strengths that generate confidence in its findings and interpretations. (1) It is the first study simultaneously measuring fNIRS neuroimaging with the key systemic physiological parameters (HR, RR, $P_{\mathrm{ET}}{\mathrm{CO}}_{2}$, MAP, and SCL) as genuine SPA-fNIRS. (2) It is also the first SPA-fNIRS study employing the PRQ parameter. (3) It is the first study that investigated the effect of a short-term colored-light exposure on both the human brain and the systemic physiology. (4) In addition, the different colors of our wide-field stimulation had equal illuminance and defined spectral characteristics. This avoids confounding effects present in several other color studies. (5) Our multidistance frequency-domain fNIRS approach^60^ ensured a great reduction of the influence of the superficial extracerebral tissue layers and MAs on the final $\left[O_{2}\mathrm{Hb}\right]$, [HHb], [tHb], and ${\mathrm{StO}}_{2}$ compared to common continuous wave (CW)-fNIRS with single distances.^62^^,^^63^ In addition, our approach provides absolute values based on spectroscopic technique employed and reasonable assumptions made concerning the determination of the photon propagation in tissue. (6) Simultaneously, measuring both the PFC and VC enabled their different responses to the colored light to be determined. (7) The repeated short-term stimulation paradigm enables responses to be assessed for statistical significance at an individual subject level. By including only signals that are significant at the subject level, we ensured that only physiologically relevant changes were included in the group analysis. Furthermore, the nonparametric statistical analysis ensured that outliers in the data did not lead to errors. Without these two measures, the signal-to-noise ratio would have been lower, such that the color-dependent effects would be masked [as confirmed by an additional analysis (data not shown)]. (8) The coupling analysis delivered additional insights into the color-dependent relationships of the physiological signals. Again, this is the first time that such an analysis has been reported for fNIRS studies.

#### 4.6.2 Limitations

This study has a few limitations. (1) A larger number of subjects than 14 may have revealed additional color-dependent responses. (2) Some subjects reported growing tiredness during the experiment. This may have diminished the effect of the colored light, but it is difficult to avoid this. (3) Systemic changes might have influenced cerebral blood circulation and thus the fNIRS signals, since the FDMD fNIRS method in the same way as CW-fNIRS, fMRI, and positron emission tomography (PET) is not able to reduce the impact of changes in systemic physiology in the brain. This may lead to false interpretation of the signals.^149^ For example, changes in $P_{\mathrm{ET}}{\mathrm{CO}}_{2}$ are a known strong confounding factor.^150^^–^^153^ The significant changes, e.g., in MAP, RR, HR, PRQ, and MWA, in our study indicate that our cerebral fNIRS signals may have been influenced by these factors. Removing these confounding effects is a challenging task and a topic of current research. This problem also affects other hemodynamic neuroimaging methods (fMRI and PET, see Sec. 4.7). (4) The coupling analysis assessing the neurosystemic functional connectivity is influenced by the structure of the correlation matrices. However, we constructed these matrices by grouping physiologically related signals (e.g., grouping all $\left[O_{2}\mathrm{Hb}\right]$ values, followed by all [HHb] values, etc.). In addition, since we primarily tested relative differences of the complex network parameters between colors, the effect of the correlation matrix structure was not relevant. (5) For the EDA, we quantified the slower SCL, because we expected changes in the same time range as hemodynamic responses. The faster SCR may provide additional insights. (6) In the future, EDA should be measured on both hands because the EDA difference between the hands (termed “EDA asymmetry”) is an additional innovative indicator for the state of the ANS^154^^–^^157^ to potentially capture reactivity to the colors. EDA was only measured on one hand, because the other hand was occupied by the pulse oximetry sensor.

### 4.7 Implications for Functional Brain Activation Studies

There are several methods that employ cerebral hemodynamics as a biomarker of brain activity. In addition to fNIRS and PET, the most widely applied today is fMRI. Our results demonstrate that even nonstrenuous, perception-based functional brain activity leads to changes in systemic physiology, in particular the MAP, which has previously been shown to influence cerebral hemodynamics. Other relevant parameters, such as HR and RR, were also changed. The latter implies that there should also have been a change in $P_{\mathrm{ET}}{\mathrm{CO}}_{2}$ in response to the color stimulations; this effect was only visible at an individual level, however, and was not significant at a group level. $P_{\mathrm{ET}}{\mathrm{CO}}_{2}$ is the parameter known to affect cerebral blood flow most strongly.^151^ These changes in several systemic physiological variables could very well be important confounders in functional activation studies using fMRI, PET, and fNIRS. Methods to disentangle the different influences on cerebral hemodynamics are urgently needed.^158^ To detect these possible confounders, an SPE approach as employed in this study is required.

## 5 Conclusions and Outlook

In conclusion, this SPA-fNIRS study demonstrates a wide variety of effects evoked by colored light: (i) the cerebral response varies with region. While the VC reacted to all colors, the PFC responded significantly only to blue light, which was significantly different to red and green light; (ii) a color-dependent effect on the cardiorespiratory activity was observed for PRQ (blue: decrease), HF (red: increase, blue and green: decrease), LF (green: decrease, red and blue: increase), LF/HF (red and blue: increase, green: decrease); (iii) the magnitude of hemodynamic responses in the PFC and the VC were gender- and age-dependent; (iv) gender and age effects were visible in the cardiorespiratory system: gender and age affected RR and PRQ; age affected LF, LF/HF, and MWA (LPFC); gender affected $P_{\mathrm{ET}}{\mathrm{CO}}_{2}$, PP, Q, and MWA (RPFC); and (v) EDA and psychological state did not show significant changes. Thus, we were able to demonstrate responses in cerebral hemodynamics/oxygenation as well as cardiorespiratory dynamics that were color dependent. Blue light was found to elicit the strongest effect overall.

Our findings contribute to a better understanding of the effects of colored light on human physiology. This is relevant both for medical research and everyday life. Colored light has already been employed as a treatment modality in medicine. This information opens opportunities to specifically treat diseases, using colored light as a potentially powerful tool for future medicine. In addition, we are increasingly exposed to colored light in everyday life (low-energy light bulbs and screens), whose potential for disease-promoting effects should be considered (recent reviews: Refs. 159–161). This is a topic for urgent further investigation.

The significant correlations between cerebral and systemic parameters demonstrate a neurosystemic functional connectivity, which has in most incidences been neglected so far. The presence of this neurosystemic functional connectivity should be taken into consideration in future neuroscientific studies and could constitute a paradigm shift. SPA-fNIRS should become a standard approach for functional neuroimaging experiments using fNIRS because even our apparently nonstrenuous stimulation of colored light significantly altered the human systemic physiology. Mentally or physically strenuous tasks are expected to elicit even more profound systemic effects (whereby the strength of these systemic effects is expected to be related to the exact kind of tasks/stimulations performed and the individual physiology), which are likely to affect cerebral hemodynamics. These effects should be explored further because the concept that cerebral hemodynamic changes are purely associated with brain activity is probably wrong. This is not only true for fNIRS but also for other hemodynamic neuroimaging methods including the most widely used neuroimaging modality fMRI.
